# Oculomotor Behavior Predict Professional Cricket Batting and Bowling Performance

**DOI:** 10.3389/fnhum.2021.768585

**Published:** 2021-12-24

**Authors:** Nicholas P. Murray, Josh Lawton, Patrick Rider, Nathanial Harris, Melissa Hunfalvay

**Affiliations:** ^1^Visual Motor Laboratory, Department of Kinesiology, East Carolina University, Greenville, NC, United States; ^2^College of Education, Florida State University, Tallahassee, FL, United States; ^3^RightEye, LLC, Bethesda, MD, United States

**Keywords:** eye-tracking, cricket, performance, regression analyses, oculomotor behavior

## Abstract

**Importance:** A new, shorter version of cricket was introduced recently (Twenty20; T20). Since its inception, T20 cricket has rapidly become a popular and exciting format of cricket. However, there is little understanding of factors such as visual-motor control that influence expert performance.

**Objective:** The purpose of this project is to determine if a series of oculomotor measures can predict batting and bowling performance in professional cricket players.

**Design:** This study used a cross-sectional design. Each participant took part in a suite of eye-tracking tests to measure oculomotor behavior compared to their performance data.

**Participants:** This study used a sample of 59 male T20 league professional cricket players (30 Bowlers and 29 Batsman).

**Results:** One-way univariate analyses of variance examined the differences in oculomotor behavior between batsman and bowlers. A series of multiple regression analyses was conducted to evaluate how well the visual variables predict bowling and batting performance variables. Results demonstrate that several oculomotor eye tracking measures were good predictors of run performance and strike rate, including sports total score, sports on-field score, and sports functional score. Likewise, several of the same metrics predicted Runs and Wicket performance for bowlers. Overall, results provided further validation to a growing body of literature supporting the use of eye-tracking technology in performance evaluation.

## Introduction

In the last few decades, motor behavior and sport psychology literature have extensively examined sport expertise’s definition and identification. This concept has been defined in multiple ways, primarily focusing on either a naturalistic approach aimed on talent identification or an environmental approach, looking at practice as the primary vehicle to reach higher levels of expertise (Janelle and Hillman, 2003). Recently, a more “interactionist” approach to look at expertise has been advanced. Although recognizing the relevance of practice, this approach considers hereditary factors, individual differences, genetic characteristics, and especially motor and sensory function that potentially limit the level of acquirable expertise (Janelle and Hillman, 2003).

Researchers have investigated optimal visuomotor strategies utilized by experts in self-paced (Janelle and Hillman, 2003), and externally paced tasks (Murray and Hunfalvay, 2017; Hunfalvay and Murray, 2018) consistently concluding how specific visuomotor strategies mark skilled behaviors; however, few studies have described the relationship between player performance and visual motor control. Previous research that relates baseball performance with eye-tracking has found that professionals with better eye tracking are less likely to swing at pitches inside and outside the strike zone (Laby et al., 2019; Liu et al., 2020a). The fewer swings at pitches inside the strike zone imply that individuals with better visual abilities are more discerning in their swings (Liu et al., 2020a). Eye-tracking skills are noticeably better in the Major League Baseball players, demonstrated by general oculomotor speed being a significant predictor of league level in baseball (Liu et al., 2020a). Research has also shown significant differences in other eye-tracking abilities, including cardinal gaze between professional baseball players versus amateurs and non-athletes (Kubitz et al., 2020). Furthermore, there are several recent reviews of visual-motor control in baseball. For example, Toole and Fogt (2021) described the relatively consistent pattern of horizontal head and eye tracking movements in baseball batting until an anticipatory saccade occurs. As such visual-motor control is a significant factor in batting performance. Although batting performance is due to factors within perception-action coupling such as perceptual knowledge, attentional mechanisms, cognitive control, and motor function, and while under some debate, recent work has also demonstrated the importance of visual-motor assessment and training in high-level sport. Likewise, superior visual abilities are found among many high-level athletes. Laby and Appelbaum (2021) provide an extensive review on the association of visual-motor control and athletic performance and describe the potential value of sports vision training.

Although there is a growing effort in baseball to understand visual-motor control; few studies have examined these variables in cricket. Early work by Land and McLeod (2000) related cricket players’ performance and visual-motor control by tracking the eyes of batsmen as a bowler pitches them a ball. The better the individual was at the sport, the quicker the athlete used anticipatory saccades to shift their eyes to where the ball would bounce (Land and McLeod, 2000). The low-level club cricket player tested was either slower to initiate a saccade or did not use a saccade at all when tracking the ball. It was determined that the speed and variability in the timing of the initial saccade are related to batting performance.

Few definitions have been presented in previous literature to define expertise in the sport of cricket. Recently, several researchers have advanced methods to improve the evaluation of cricket skills. Studies have analyzed past performances to predict future outcomes, including a model that correctly predicts 71% of test cricket outcomes. Brooks et al. (2002) used data from 1994 to 1999 to create an ordered response model that can predict a match loss correctly 81% of the time, compared to predicting a match draw 57% of the time. The data used includes performance and strike rate for both batting and bowling coefficients. These investigators then ran through which predictions the model failed and found that different locations were more predictable than others, as Sri Lanka has a higher tendency to be involved in predictable matches. In contrast, Pakistan was less likely to be predicted correctly. The study found that there are five different styles of predicting outcomes. Each style describes one country, and some represent a second country to a lesser extent. The authors specified the predictability for each country studied and identified poor predictors of which the three most common are unsuccessful last innings runs chase; successful last innings runs chase, and weather-affected matches.

In 2003, a new, shorter version of cricket was introduced, labeled Twenty20 (T20). Since its inception, T20 cricket has rapidly become the most lucrative and desirable format of the game (Irvine and Kennedy, 2017). Due to the increasing popularity of the sport, there has been a wave of new research investigating successful tactics and strategies to facilitate perform better. The total number of dot balls bowled, the total number of wickets taken, and the innings run rate were the most significant indicators of success (Irvine and Kennedy, 2017). The findings show that batting sides should look to maximize their run rate per over throughout the entire inning, emphasizing selecting a batsman with high strike rates and supporting the notion of batting index (average + strike rate). These indicators of success remain consistent across different cricketing environments, with scoring and batting generally higher in sub-continent conditions.

Sharma (2012) also examined performance in T20 by conducting a factor analysis to determine if batting capability is superior to bowling capability. Sharma examined 85 batters and 85 bowlers from the Indian Premier League (IPL) with the following statistics for the batters: individual score, average batting performance, strike rate, numbers of fours and sixes, and for bowlers: economy rate (ECON), bowling average, and bowling strike rate. The study found that the variance explained by batting was 48.51%, and the variance explained by bowling was 20.23%. The variance of batting being much higher than the variance of bowling proves the higher importance of batting to bowling in T20 cricket (Sharma, 2012). While these statistics demonstrated differences between expertise, it can be challenging to differentiate the individual and team success. Manage, Scariano, and Hallum (Manage et al., 2013) analyzed the T20-World Cup Cricket 2012 data with a multivariate statistical analysis to rank the batsmen. The investigators used innings, runs, average, and strike rate to determine a point system, called MR points, to quantify contributions of cricket batters. The investigators compared matches, wickets, average, and ECON for bowlers to determine their MR points. Also, the authors calculated the players’ FPC rank, consisting of overs, wickets, average, ECON, and strike rate. The findings found that the MR method and FPC method had 8 of the top 10 batters and 8 out of the top 10 bowlers the same, showing that the FPC method is a transparent and straightforward way to evaluate cricket players with results comparable to previous methods.

Although several studies have examined visual behavior and baseball performance, few have examined Cricket performance, especially considering the recent advances in performance metrics. In addition, there is little research conducted specifically on the oculomotor control of professional athletes. Further investigation is also necessary to challenge the long-standing hypothesis that professional athletes lack superior visual behavior and that performance is solely related to relevant skill acquisition. Therefore, the primary purpose of this study was to determine if a series of oculomotor measures can predict batting and bowling performance in professional cricket players.

## Materials and Methods

### Participants

Fifty-nine T20 league cricket athletes (30 Bowlers and 29 Batsman) were selected for this study. All participants were current members of professional teams in either the Big Bash League or South Australian Cricket Association. The stats for these players included their career T20 stats and were acquired online from ESPNcricinfo.com. The bowlers performance statistics, including innings, balls, runs, wickets and ECON (Runs/Overs bowled), are found in Table 1, and average batting and fielding statistics (Innings, Balls Faced (BF), Strike Rate (SR), X4’s, X6’s, Centuries, and Catches) for Batsman are found in Table 2.

**TABLE 1:** Average (SD) bowlers performance statistics.

Bowling				

**Innings**	**Balls**	**Runs**	**Wickets**	**ECON**
49.38 (50.96)	999.63 (1101.92)	1353.63 (1474.44)	54.63 (63.72)	9.48 (3.27)

**TABLE 2:** Average (SD) batting and fielding performance statistics.

Batting and Fielding						

**Innings**	**Balls faced**	**Strike rate**	**X4’s**	**X6’s**	**Centuries**	**Catches**
33.44 (31.94)	1671.40 (1938.10)	65.20 (58.30)	189.90 (231.29)	85.00 (107.47)	0.30 (0.94)	51.20 (67.049)

Participants were excluded from participation in the study if they met any of the following pre-screening conditions: neurological disorders (such as concussion, traumatic brain injury); vision-related issues that prevented successful calibration (Thiagarajan et al., 2011; Bellmann et al., 2014) of all 9-points [such as extreme tropias, phorias (Thompson et al., 2006), static visual acuity of greater than 20/400 (Bellmann et al., 2014), nystagmus,(Thompson et al., 2006; Bellmann et al., 2014), cataracts (Hunfalvay et al., 2020) or eyelash impediments (Hunfalvay et al., 2020)]; consumption of drugs or alcohol within 24 h of testing. All participants provided informed consent to participate in this study in accordance with IRB procedure. All testing was conducted by vision specialists (e.g., optometrists, ophthalmologists) and had received and passed the RightEye training, education, and protocol procedures prior to testing.

#### Materials and Equipment

During the RightEye test, the participants were seated in a stationary (non-wheeled) chair that could not be adjusted in height at a desk within a quiet, private testing room. The participants were asked to look at a NVIDIA 24-inch 3D Vision monitor that could be adjusted in height which was fitted with an SMI 12″ 120 Hz remote eye tracker connected to an Alienware gaming system, and a Logitech (model Y-R0017) wireless keyboard and mouse. Screen luminance was 85cd/m2, room luminance with the lights on was 344cd/m2. Participant’s heads were unconstrained during the test, although they were instructed to sit still.

The eye tracker is used to capture the x and y coordinates for each eye, along with the z-distance at 120 times a second.

##### Oculomotor Testing Tasks

The Functional Vision EyeQ model (FVEQ) includes a linear combination of saccade, pursuit, fixation, and reaction time oculomotor variables. A total of 58 metrics make up the FVEQ. Weights range from 0.1 to 13% across metrics. Based on our previous work (Murray and Hunfalvay, 2017) and work of others (Burris et al., 2018) only the following metrics were analyzed (see Table 3):

**TABLE 3:** Visual motor metrics.

Variable	Definition
Brain overall score	A measure of fixations, saccades and smooth pursuit eye movements
Brain fixation score	A measure of the ability to keep the eyes still (fixate)
Brain fixations percentile	Compares the ability of the person to fixate compared with normative values
Brain pursuits score	A measure of smooth pursuit eye movements
Brain pursuits percentile	A measure of the ability to smooth pursuit compared with normative values
Brain saccades score	A measure of the ability to conduct saccadic eye movements
Brain saccades percentile	Compares the ability of the person to saccade compared with normative values
Sports total score	A collection of functional, mechanics, mind-eye and on-filed scores
Sports on-field score	A measure of visual on/off-task time
Sports MindEye score	A measure of processing and reaction time in choice and discriminate tasks
Sports mechanics score	A measure of oculomotor metrics (fixations, saccades, and smooth pursuits)
Sports functional score	A measure of acuity (static and dynamic), and contrast sensitivity

*Functional* refers to the ability to have basic, fundamental visual health and functionality such as acuity, contrast, dryness.
*Mechanics* refers to the eyes teaming together and muscle and nerve coordination to maintain effective and efficient use of the eyes.
*Mind-Eye* refers to the interplay of vision and neuro-connectivity to include visual processing.
*On-Field* refers to how environmental factors can influence performance including vision via motor responses such as reactions, impulses and distractibility.
*Sports Total score* is a model based logistic regression score.

#### Testing Procedure

Participants were recruited through professional cricket teams. The study was conducted in accordance with the tenets of the Declaration of Helsinki. The nature of the study was explained to the participants, and all participants provided written consent to participate. Following informed consent, participants were asked to complete a pre-screening questionnaire and an acuity vision screening where they were required to identify four shapes at 4 mm in diameter. If any of the pre-screening questions were answered positively and any of the vision screening shapes were not correctly identified, then the participant was excluded from the study. Qualified participants who successfully passed the nine-point calibration sequence completed the eye-tracking tests. The calibration sequence required participants to fixate one at a time on nine points displayed on the screen. The participants had to successfully fixate on at least eight out of nine points on the screen to pass the calibration sequence. Written instructions on screen and animations were provided before each test to demonstrate appropriate behavior required in each test.

### Data Analysis

A preliminary analysis was first performed to check for violations of statistical assumptions. First, we compared oculomotor variables by position with separate univariate ANOVAs. Next, we conducted a series of multiple regression analyses to evaluate how well the visual variables predict bowling and batting performance variables. The predictors were batting indices (Runs and Strike Rate) and bowling performance indices (Balls, Runs, Wickets, and ECON), while the criterion variables were oculomotor measures. Runs represent the number of runs scored, and strike rate is the average number of runs scored per 100 balls faced. A higher strike rate represents how effective a batsman is at scoring quickly. ECON indicates the average number of runs conceded per over (i.e., Econ = Runs/Overs bowled). The data were analyzed using the Statistical Package for the Social Sciences software (SPSS) version 23.0 (SPSS, Chicago, IL, United States). A value of *p* < 0.05 was considered statistically significant, and when appropriate we used Bonferroni adjustments for *p*-value.

## Results

### Descriptive Comparison by Position

Using separate univariate ANOVAs, we compared oculomotor variables by position. There were no significant differences by position for all oculomotor variables (see Table 4).

**TABLE 4:** Mean comparison of oculomotor variables by position.

	Batsman	Bowler	Sig.
Brain overall score	86.45 (10.41)	81.92 (14.71)	0.316
Brain fixation score	70.3 (20.51)	72.42 (25.05)	0.796
Brain fixations percentile	72.55 (21.55)	74 (25.69)	0.856
Brain pursuits score	81.2 (11.84)	71.83 (20.95)	0.115
Brain pursuits percentile	94.15 (13.91)	84.33 (28.43)	0.199
Brain saccades score	61.15 (11.01)	64.58 (8.52)	0.363
Brain saccades percentile	75.4 (17.27)	80.83 (12.36)	0.349
Sports total score	70.05 (5.24)	70.5 (3.26)	0.794
Sports on-field score	87.63 (8.42)	87.17 (9.49)	0.888
Sports MindEye score	42.79 (8.82)	43.25 (12.43)	0.905
Sports mechanics score	71.89 (5.05)	73.92 (4.76)	0.276
Sports functional score	78.37 (7.85)	77.33 (5.68)	0.696

### Regression Analysis Batting Statistics

For Batting statistics, a stepwise multiple regression of brain overall score, brain fixation score, brain fixations percentile, brain pursuits score, brain pursuits percentile, brain saccades score, brain saccades percentile, sports total score, sports on-field score, sports mindeye score, sports mechanic score, and sports functional score was performed to predict Runs. The linear combination of the RightEye variables was significantly related to Run performance, *F*(10,27) = 5.727; *p* < 0.001. Overall, 68.1% of the variance in runs was explained by this model, *R*^2^ = 0.68. Sports total score, sports on-field score, and sports functional score were further considered because they showed significance and were not highly correlated with other variables (see Table 5). Brain overall score and brain fixations percentile were removed from the analysis because these variables were highly correlated with other variables. The results revealed that sports total score, *t*(−1.784) = −3.163, *p* = 0.027, sports on-field score, *t*(2776.894) = 3.045, *p* = 0.006, and sports functional score, *t*(949.107) = 2.443, *p* = 0.024, were significant predictors to the number of Run performance.

**TABLE 5:** Correlation statistic between variables for batsmen.

Variable	1	2	3	4	5	6	7	8	9	10	11
1 Brain Overall Score											
2 Brain Fixation Score	–0.002										
3 Brain Fixations Percentile	–0.003	0.874*									
4 Brain Pursuits Score	0.811*	–0.130	–0.127								
5 Brain Pursuits Percentile	0.912*	–0.151	–0.148	0.926							
6 Brain Saccades Score	0.529*	–0.467	–0.474	0.184	0.283						
7 Brain Saccades Percentile	0.537*	–0.459	–0.465	0.196	0.289	0.996*					
8 Sports Total Score	0.608*	0.382	0.383	0.430	0.511	0.129	0.123				
9 Sports On-field Score	0.392	0.296	0.297	0.181	0.340	0.010	–0.005	0.879*			
10 Sports MindEye Score	0.181	0.235	0.234	0.078	0.076	0.116	0.097	0.775*	0.651		
11 Sports Mechanics Score	0.711*	0.028	0.026	0.688	0.637	0.374	0.382	0.416	0.244	–0.062	
12 Sports Functional Score	0.411	0.594	0.598	0.198	0.350	–0.184	–0.172	0.759	0.675	0.563	–0.034

*p < 0.05.

The second model was significant indicated by *p* < 0.001. Overall, 85% of the variance in Strike Rate was explained by Sports Mechanics Score, Brain Pursuits Score, Sports MindEye Score, Brain Saccades Score, Brain Fixation Score, and Sports On-field Score, *R*^2^ = 0.85 (see Table 6).

**TABLE 6:** Model characteristics for SR.

	Beta	Std. Error	*t*	Sig.
(Constant)	722.484	12.056	59.925	0.000
Sports mechanics score	15.547	0.240	–64.894	0.000
Brain pursuits score	2.639	0.113	23.293	0.000
Sports MindEye score	3.024	0.137	22.037	0.000
Brain saccades score	1.602	0.124	12.929	0.000
Brain fixation score	0.356	0.049	7.347	0.000
Sports on-field score	0.572	0.100	–5.724	0.001

### Regression Analysis Bowling Statistics

A stepwise multiple regression of brain overall score, brain fixation score, brain fixations percentile, brain pursuits score, brain pursuits percentile, brain saccades score, brain saccades percentile, sports total score, sports on-field score, sports mindeye score, sports mechanic score, and sports functional score was performed to predict Runs. The model was significant indicated by *p* = 0.032. Overall, 45.5% of the variance in runs was explained by this model, *R*^2^ = 0.455.

Brain overall score, brain pursuits percentile, and brain saccades percentile were further considered because they showed significance. The results revealed that the brain overall score, *t*(−4673.655) = −3.163, *p* = 0.005, brain pursuits percentile, *t*(2776.894) = 3.045, *p* = 0.006, and brain saccades percentile, *t*(949.107) = 2.443, *p* = 0.024, were significant predictors to the number of Runs achieved by the athletes (see Table 7).

**TABLE 7:** Correlation statistic between variables for bowlers.

Variables	1	2	3	4	5	6	7	8	9	10	11
1. Brain overall score											
2. Brain fixation score	–0.317										
3. Brain fixations percentile	–0.292	0.968*									
4. Brain pursuits score	0.977*	–0.416	–0.39								
5. Brain pursuits percentile	0.968*	–0.484	–0.46	0.983*							
6. Brain saccades score	–0.306	–0.002	–0.015	–0.377	–0.433						
7. Brain saccades percentile	–0.311	–0.008	–0.021	–0.387	–0.436	0.996*					
8. Sports total score	–0.026	–0.17	–0.164	0.1	0.016	–0.044	–0.034				
9. Sports on-field score	0.614*	–0.469	–0.451	0.737	0.678	–0.319	–0.352	0.567			
10. Sports MindEye score	–0.277	–0.192	–0.194	–0.201	–0.173	–0.175	–0.119	0.609	0.069		
11. Sports mechanics score	–0.079	–0.091	–0.093	–0.005	–0.121	0.461	0.41	0.384	0.355	–0.333	
12. Sports functional score	–0.255	0.673	0.671	–0.334	–0.382	0.084	0.093	0.069	–0.397	–0.18	–0.009

*p < 0.05.

To predict Wickets, stepwise multiple regression of brain overall score, brain fixation score, brain fixations percentile, brain pursuits score, brain pursuits percentile, brain saccades score, brain saccades percentile, sports total score, sports on-field score, sports mindeye score, sports mechanic score, and sports functional score, was performed. The model was significant indicated by *p* = 0.002. Overall, 84.6% of the variance in runs was explained by this model, *R*^2^ = 0.846. Lastly, Wickets was considered and as there was significance when related to brain overall score, brain pursuits percentile, brain saccades score and percentile, sports total score, sports on field score, sports mind eye score, sports mechanics score and sports functional score. The results revealed the significant predictors for the amount of Wickets are the brain overall score, *t*(131.349) = 5.665, *p* = < 0.001, brain pursuits percentile, *t*(−80.376) = −3.166, *p* = < 0.001, brain saccades score, *t*(−21.485) = −3.166, *p* = 0.07, brain saccades percentile, *t*(−19.549) = −2.986, *p* = 0.011, sports total score, *t*(82.882) = 3.960, *p* = 0.002, sports on field score, *t*(−12.373) = −3.748, *p* = 0.002, sports mind eye score, *t*(−18.727) = −3.841, *p* = 0.002, sports mechanics score *t*(−27.796) = −3.633, *p* = 0.003, and sports functional score *t*(23.417) = −4.210, *p* = 0.001.

Stepwise multiple regression with the same variables was performed to predict Econ. The model was not significant indicated by *p* = 0.726. Overall, 39.4% of the variance in runs was explained by this model, *R*^2^ = 0.394. Econ was not selected for further consideration as there was no significance when related to eye tracking results.

## Discussion

The interceptive task of hitting in cricket is a strikingly challenging task. This alone presents a level of difficulty that would surpass the coordination skills of many individuals. Vision training in other sports such as baseball has decreased the required reaction time for success and correlates to increased hitting performance (Kohmura and Yoshigi, 2004; Maman et al., 2011; Clark et al., 2012). However, as vision training is contemporary, its relationship to sports performance is still controversial (Knudson and Kluka, 1997; Wood and Abernethy, 1997; Khanal, 2015). The primary purpose of this study was to determine if a series of oculomotor performance can predict batting and bowling performance in professional cricket players. Results demonstrate that several oculomotor eye-tracking measures were good predictors of run performance and strike rate. In particular, batsman with better oculomotor function and oculomotor mechanics tended to have higher run scores and higher strike rate. These results support the hypothesis that better oculomotor skills predict better batsman performance. Likewise, several of the same metrics predicted Runs and Wicket performance for bowlers. Specifically, we found that brain overall score, brain pursuits percentile, and brain saccades percentile were predictors of Runs performance, and similarly, these measures were predictors of Wicket. Successful bowlers integrate sensory and motor functions, including proper mechanics and visual control to a target location. Combined, this represents a critical link between oculomotor behavior and motor performance.

Similar to Liu et al. (2020a), the present study demonstrates that oculomotor function is related to cricket performance. Most interestingly, eye tracking measures that indicate fundamental health (functional) and motor and nerve coordination (mechanics) have predictive potential to strike rate and runs (*R*^2^ = 0.85). On-Field score, which includes reactivity, impulsivity and distractibility, and saccadic behavior, also demonstrated high predictive value for batting performance. These findings align well with multiple studies that have correlated better batting performance with earlier longer and more anticipatory saccade movements (Land and McLeod, 2000; Mann et al., 2013). For example, Kruger et al. (2010) found that training higher-level cricket players’ eyes lead to significant improvement in advanced ball skills, pursuit eye movements, eye tracking, visual anticipation, accuracy, and more. Finding the eye movement characteristics that help improve hitting success in players can lead to development/training programs that will enhance gaze pattern abilities in cricket players. Breaking down the many components of gaze patterns and finding the ones that influence hitting success is vital in creating a gaze pattern development or training program focused on improving hitting. In baseball, recent research has found visual acuity differences between baseball players and non-baseball players at the youth level (Boden et al., 2009). Also, studies have shown that functional factors, such as dynamic visual acuity (DVA), develop with age in both males and females (Kohmura et al., 2008). While there are already physical training programs aimed toward youth athletes (Szymanski, 2013), functional vision training programs could be implemented at the youth level to create future success. Boden et al. (2009) found that youth baseball and softball players had better static stereo acuity than non-ball players. Their finding suggests that visual differences can be seen at an early playing age, and it may be due to the visual requirements needed to be successful in hitting a moving ball. Vision training programs have been utilized to enhance stereopsis and sensorimotor abilities in baseball/softball settings (Clark et al., 2015; Appelbaum et al., 2016). DVA training programs have been used at the college level with some success (Kohmura and Yoshigi, 2004; Clark et al., 2012; Deveau et al., 2014). For example, Clark et al. (2015) implemented standard vision training exercises with a university baseball team for 2 years and found that the team improved their hitting statistics overall year over year. In addition, more recent studies using pre-post treatment-control designs have demonstrated increased performance following perceptual–cognitive skills training. Gray (2017) demonstrated positive transfer of training in pitch recognition within a virtual environment (VE). Specifically, Gray examined 4 different training protocols, including a control group, and found that the adaptive VE training group (increased challenged with correct responses) had significantly better batting performance than other conditions. Similarly, Liu et al. (2020b), in a placebo-controlled trial with a modified perceptual training framework, demonstrated that dynamic vision training had positive transfer to sport-specific batting practice performance, however, noted that there was not yet transfer to game performance. Along with Lui et al’s work, Gray, and this current study lead to the potential of perceptual–cognitive skills training and transfer to game performance.

### Future Directions and Limitations

There were some limitations associated with this study; however, generally, these limitations lead to important future directions. The data described here is exploratory in nature, and outcomes were based on effect size, goodness-of-fit, and most theoretically interpretable. The r-square of 0.85 within the strike rate model represents how well the model fits the data. Although there is potential to overfit the model; we accounted for this by removing variables with high multicollinearity and utilizing highly predictable variables that influence strike rate performance. Clearly, eye movement assessment is not the only predictor of performance, and this finding should be considered with some caution; however, it does illuminate a close link between visual-motor control and performance.

Furthermore, it may be possible that a more fine-tuned assessment of oculomotor behavior is necessary to capture the sensory and motor components of batting and bowling. For example, a more in-depth analysis of smooth pursuit velocities and gaze error given a target position to name a few. This work and work by others, including Burris et al. (2018) and Laby and Appelbaum, have demonstrated clearly that visual ability influences motor performance in interceptive tasks such as baseball and cricket. High-performing athletes demonstrate that both heredity and environmental conditions contribute to success. This recent work indicates that there are both functional and training differences that influence motor performance success. Further research is necessary to determine what training strategies can facilitate batting success and to fully understand the relationships between the sensory and motor components of cricket performance. The goal of training should be to limit the required reaction time for success by training to enhance the sensory components of the batting and bowling process.

## Conclusion

The aims of this study were to examine the relationship between eye movement patterns and batting and bowling performance in cricket. The results demonstrated that several oculomotor eye-tracking measures were good predictors of batting and bowling performance in cricket. Additional research is needed to provide a more thorough understanding of the relationship between oculomotor function and performance and develop effective training methods that improve these swing qualities, which are indicative of skill level and success rates.

## Data Availability Statement

The original contributions presented in the study are included in the article/supplementary material, further inquiries can be directed to the corresponding author.

## Ethics Statement

The studies involving human participants were reviewed and approved by the East Carolina University IRB. The patients/participants provided their written informed consent to participate in this study.

## Author Contributions

All authors contributed to report writing, data analysis, and data collection.

## Conflict of Interest

MH is the Chief Science Officer & Co-Founder of RightEye, LLC. The remaining authors declare that the research was conducted in the absence of any commercial or financial relationships that could be construed as a potential conflict of interest.

## Publisher’s Note

All claims expressed in this article are solely those of the authors and do not necessarily represent those of their affiliated organizations, or those of the publisher, the editors and the reviewers. Any product that may be evaluated in this article, or claim that may be made by its manufacturer, is not guaranteed or endorsed by the publisher.
